# DE-HRNet: Detail enhanced high-resolution network for human pose estimation

**DOI:** 10.1371/journal.pone.0325540

**Published:** 2025-09-02

**Authors:** Yuxuan Liu, Guohui Zhou, Wei He, Hailong Zhu, Yanling Cui

**Affiliations:** School of Computer Science and Information Engineering, Harbin Normal University, Harbin, China; University of Electronic Science and Technology of China, CHINA

## Abstract

Scale variation is a challenge in human pose estimation. The scale variations of human body are related to the accuracy and robustness of posture estimation. For example, the prediction accuracy of smaller joints (such as ankles and wrists) is less than that of larger joints (such as head and shoulders). To address the impact of scale variations across parts of the human body on the positioning of key points. In this paper, we propose a Detail Enhanced High-Resolution Network (DE-HRNet), which can efficiently extract local detail features and mitigate the impact of scale variations for human pose estimation. First, we propose a Detail Enhancement Module (DEM) to relearn the lost low-level detailed features and enhance the model’s ability to capture delicate local features, which is crucial for improving the accuracy of scale-varying keypoints. Second, we introduce an ultra-lightweight dynamic sampler - dySample, which is used to replace nearest up-sampling. It aims to reduce the loss of detail information from low-resolution features during up-sampling, while simultaneously preserving finer local representations for high resolution, it can be beneficial in improving the robustness of the model in dealing with scale-varying keypoints. On the COCO test-dev2017 and MPII valid datasets, our method achieved 75.6 AP and 90.7 PCKh@0.5, respectively, compared to High-Resolution Network (HRNet), it improved by 0.7 and 0.4 points. In comparison with the other works, the proposed method has performed well in the scale variation.

## 1 Introduction

2D human pose estimation is a challenging task in computer vision, providing crucial technical support for downstream vision tasks such as pose tracking, action recognition, and action prediction. However, as a pivotal component of pose estimation, keypoint localization often faces persistent challenges in practical scenarios due to factors including scale variations [1,2], illumination changes, occlusions, and complex backgrounds, which collectively hinder the achievement of robust performance.

Scale variation presents a fundamental challenge in human pose estimation due to the inherent diversity of target human joints across practical scenarios. Human joints exhibit multi-scale properties where finger joints (3–5px in 640 × 480 images) require 10 × higher localization precision than torso joints [3]. These intrinsic challenges are exacerbated in real-world scenarios where camera-subject distances (5-50m), dynamic postures (e.g., crouching vs. jumping), and inter-person occlusions create compound scale variations within single frames.

**Fig 1:**
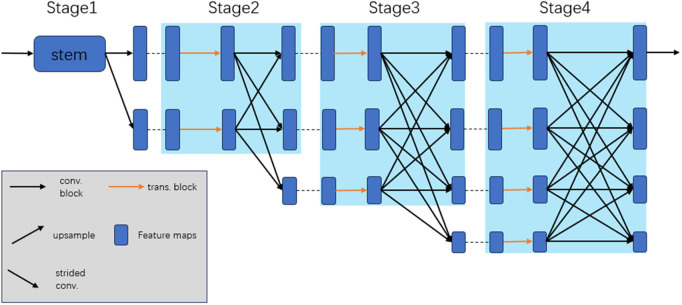
The HRNet architecture. The multi-resolution stage modules are marked with blue color areas. The remained three stages module consists of parallel multi-resolution subnetworks with multi-resolution information interactions.

Recent studies [4] have demonstrated that top-down methods, which typically localize keypoints at a single approximate scale, offer advantages in dealing with the varying scale of human joints. For example, Sun et al. [5] proposed a High-Resolution Network (HRNet), which extracts fine local representations through multi-resolution parallel interactions and preserves high-resolution representations to maintain accurate spatial information—as shown in Fig 1. The network has achieved good performance, providing a strong baseline for addressing the scale variation of human pose. HRFormer [6] is used for dense prediction, which enhances robustness in handling scale variations. In particular, the scale-aware HRNet variant has also achieved a breakthrough, such as HigherHRNet [4] and SWAHR [7]. In addition, Cai et al. [8] proposed a Residual Steps Network (RSN), which aims to effectively aggregate features of the same spatial size (intra-level features) to obtain fine local representations, the network is advantageous for identifying joints with scale variation. However, the RSN is too large in terms of network scale, which brings high resource costs and is not conducive to improving efficiency.

Unlike bottom-up methods that focus on learning scale-aware representations, and different from using intra-level feature fusion to obtain fine local representations, our method utilizes detailed information from high-resolution features, which further captures the local detail features that are lost in low-resolution images, thereby improving the model’s ability to capture local detail features. Inspired by HRNet [5,9,10,11], we find that the repeated downsampling results in the loss of detail information, which means that the model cannot capture more features of small joints. Based on this, we propose a Detail Enhancement Module (DEM) that introduces low-level detail information to achieve across channels and resolutions interaction of high-level features, thereby enhancing the ability of model to capture local details. Furthermore, we introduce an ultra-lightweight dynamic sampler – (dySample) as a replacement for nearest up-sampling. This approach effectively reduces the problem of information loss during the up-sampling process and provides a richer set of local and global feature information for inter-layer fusion. In conclusion, our method employs HRNet as the backbone, with the primary objective of addressing scale variability by enhancing the precision of predictions at keypoints across various scales. Our contributions can be summarized as follows:

1. We propose a detail-enhanced high-resolution network (DE-HRNet). It derived from HRNet, we introduced dySample to reduce information loss during the upsampling process, thereby improving the performance of the network in dealing with keypoints of scale variations.
2. We propose the Detail Enhancement Module (DEM), which effectively combines detail information from disparate resolution branches and enhances the feature representation of these branches. It refines the local representation of features by modeling channel-wise features.
3. Experimental results demonstrate that our proposed method achieves improved accuracy on the COCO and MPII datasets while maintaining comparable performance to HRNet.

## 2 Related work

### 2.1 Top-down methods

In human pose estimation, top-down methods [5,8,9,12–14] provide a fundamental basis for studying the scale variations of human keypoints when detecting objects in input images, as the keypoints detected on the human body are at an approximate scale. Consequently, we adopt a top-down approach to locate joints of different scales, which can mitigate interference from varying human scales in multi-person scenes.

### 2.2 Feature fusion and up sample

Most existing human pose estimation methods adopt the approach of inter-layer feature fusion [5,9,15]. Inter-layer feature fusion can integrate more spatial and semantic information. For example, Alejandro et al. [15] proposed a symmetric topological convolutional neural network where low-level features are upsampled in a single stage and then concatenated with high-level features. The representative method for inter-layer feature fusion is FPN [16], which fuses upsampled high-level features with low-level features through element-wise summation. The process of feature fusion is as follows:


$$\begin{array}{c} Y^{l}={ℱ}^{UP}\left(Y^{l+1}\right)+\;X^{l} \end{array}$$ (1)

Where ${ℱ}^{UP}$ denotes upsampling, $X^{l}$ 和 $Y^{l}$ represent high-resolution and low-resolution features before fusion, respectively. The results of inter-layer fusion are relatively coarse. For example, simple upsampling operations can lead to fused features that lack fine local details. In addition, HRNet [5,9] provides high-frequency local details to low-resolution features through the downsampling of low-level features. The process is as follows:


$$\begin{array}{c} Y_{i}^{l}=\;\sum_{i=0}^{n}{ℱ}^{UP}\left(Y_{i}^{l+1}\right)+{ℱ}_{down}\left(Y_{i}^{l-1}\right)+\;X^{l} \end{array}$$ (2)

Where n  denotes the resolution branches of ${stage}_{3}$ or ${stage}_{4}$. Considering that important detail information may be lost during the upsampling process. In this work, the nearest up-sampling is substituted with an ultra-lightweight dynamic sampler - dySample [17], which retains more spatially sensitive detail information and improve our sampling efficiency.

From [17], We observe that dySample is designed from a point sampling perspective to guide high-resolution feature learning. With only minimal parameter increase, it outperforms standard upsampling methods like Nearest and Bilinear in accuracy. In addition, While alternative upsampling methods like FreqFusion [18], A2U [19], SAPA-B [20] achieve competitive performance, our evaluation based on parameter count (Params) and computational complexity (FLOPs) in [18] demonstrates that dySample provides the best cost-effectiveness among these approaches.

In a word, dySample can effectively mitigate the impact of these issues, leading to a more robust and consistent upsampling process.

### 2.3 Channel attention mechanism

**Fig 2:**
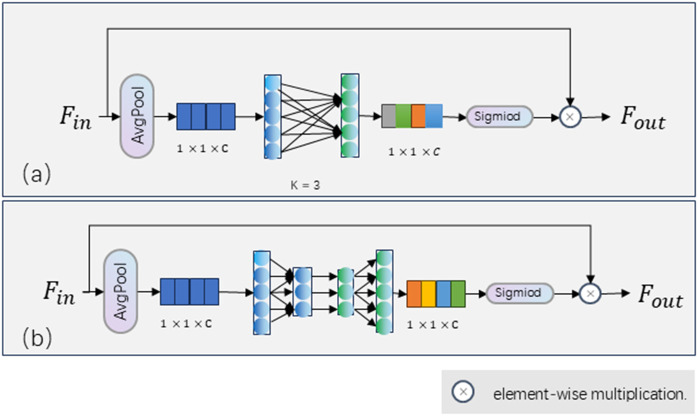
The channel attention mechanism. The top block (a) is ECA block, which consists of average pooling, a fast 1D convolution of size k and a sigmoid activation. The bottom block (b) is SE block, which consists of average pooling, two fully-connected (FC) layers and a sigmoid activation.

Channel attention mechanisms [21–23,24] aim to enhance the model’s capacity to represent specific task objectives by modeling relationships between feature channels. Hu et al. [21] proposed a SENet, as shown in the bottom block of Fig 2, which explicitly models the interdependencies between channels by leveraging global information from features. However, Wang et al. [23] argue that the dimensionality reduction of channels in SENet is detrimental to learning channel relationships and is inefficient. Therefore, they proposed an ECANet, as shown in the top block of Fig 2, which circumvents dimensionality reduction and achieves the capture of local cross-channel interaction information through one-dimensional convolution. For spatial position-sensitive tasks such as human pose estimation, the spatial positions of keypoints must have strong local feature representations. Inspired by [21,22], we propose a Detail Enhancement Module (DEM), which incorporates the SE block to learn the relationships between channels of resolution features, enhancing the capability to capture detailed information and improving the representational capacity of local features in each resolution branch.

## 3 Method

**Fig 3:**
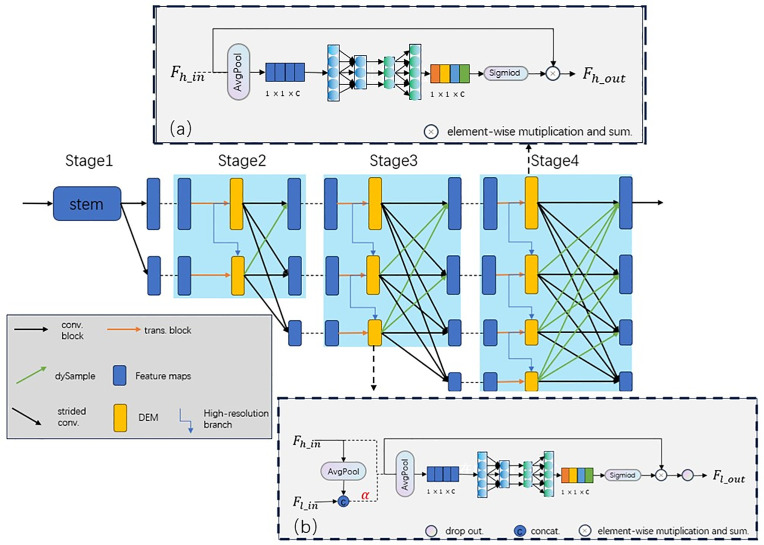
The DE-HRNet structure. The dySample and the Detail Enhancement Module (DEM) are applied to the HRNet [5,9], further implementation details are provided in Section 3.1.

The architecture of DE-HRNet is illustrated in Fig 3. Our method extends the HRNet [5,9] backbone network (the HRNet architecture as shown in Fig 1). In this section, we first provide a brief introduction to DE-HRNet. Following that, we explain our proposed DEM. In addition, we explained our proposed method and discussed the differences between our and other methods.

### 3.1 Overview of DE-HRNet

The overall network structure of DE-HRNet is shown in Fig 3, which uses HRNet as the backbone network. HRNet [5,9] consists of four stages of parallel subnetworks, the first stage only contain high resolution subnetwork, which is constructed with convolution block. The 2nd, 3rd, 4th stages consist of parallel high-to-low resolution subnetworks, which is constructed with convolution block and multi-resolution fusion.

We propose a Detail Enhancement Module (DEM) to strengthen feature representations across multi-resolution branches, which systematically improves multi-scale fusion performance through cross-branch attention mechanism. As shown in Fig 3, this module is embedded after the feature extraction stages (second, third, and fourth) but prior to multi-resolution feature fusion. For the highest-resolution branch, (a) is adopted to enhance feature representation, which employs a single input and a single output. For other branches, (b) is utilized, featuring dual inputs (from high and low-resolution branches) and producing an output for the low-resolution branch.On the other hand, in order to address the cross-resolution feature utilization gap, we integrate a content-aware adaptive sampler - dySample [17] that preserves critical structural information during upsampling through learnable kernel prediction, enabling lossless propagation of enhanced low-resolution features to guide high-resolution refinement.

### 3.2 Detail enhancement module (DEM)

**Fig 4:**
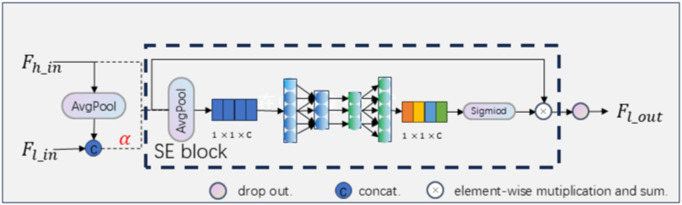
The structure of Detail Enhancement Module. The module is designed based on SE block. Additionally, the global average pooling and dropout [25] technology are located before and after the SE block, respectively. Dashed lines denote selective identity mappings.

In our study, the Detail Enhancement Module (DEM), which is primarily designed to enhance the capacity of each resolution branch to capture detailed features, thereby enhancing the network’s ability to perceive keypoints at varying scales. The structure of the DEM structure is illustrated in Fig 4. Suppose $F_{h\_in}$ and $F_{l\_in}$ are respectively the higher and lower resolution input of the DEM, $F_{l\_out}$ is the output of the DEM. The DEM contains two branches corresponding to higher and lower resolution feature, the output of higher resolution branch is high resolution feature, the output of lower resolution branch through combine higher and lower resolution feature to capture the delicate local representation.

The model evolution process is divided into two steps: Frist, the high-resolution branch goes to the next stage by identity mapping. For the low-resolution branch, we use adaptive average pooling for the low-level features, so as to preserve the semantic relationships of the low-level features, and then concatenate high-level and low-level features to get a feature map α. Second, Similar to SENet [21], we have calculated spatial weights for each resolution position to re-calibrate the different feature channels and refines the local feature information. The above generation process is formulated as:


$$\begin{array}{c} F_{h\_out}=\left(SEA\left(F_{h\_in}\right)+1\right)⊙\;F_{h\_in} \end{array}$$ (3)

$$\begin{array}{c} F_{l\_out}=dropOut\left(\left(SEA(\alpha )+1\right)⊙\;\alpha \right) \end{array}$$ (4)

Where ⊙ denotes the element-wise mutiplication, dropOut(·) [25] is used to prevent overfitting,SEA(·) is the channel weighting function:


$$\begin{array}{c} SEA(x)=\;Sigmoid\left(FC\left(RELU\left(FC\left(GAP(x)\right)\right)\right)\right) \end{array}$$ (5)

For the output of each layer of HRNet’s resolution branch, useful local features are extracted through a consistent residual block. Each layer extracts only the local features of the current branch and lacks the detailed features of the lower layers to capture finer local representations. Therefore, to address this lack of detailed features, the detail enhancement module aims to enhance the ability to capture detailed features in the resolution branch. In particular, we use a global average pooling operation to introduce detail information in high-resolution features to boost weight learning while preserving the semantic relationships between neighboring features.

### 3.3 Discussions

We use HRNet as the backbone network and our DEM with SE block as the backbone component. Therefore, we discuss the differences in our methods from two aspects:

Differences with HRNet [5]. Both HRNet and our method maintains high-resolution representations through the whole process. But they differ in the following aspects: HRNet achieves multi-resolution fusion through upsampling and downsampling across parallel resolution streams. In contrast, our method employs a DEM to augment fine-grained information in each parallel resolution branch while replacing nearest-neighbor upsampling with dySample. This design ensures that enhanced details are effectively fused across resolutions, optimizing feature preservation during cross-scale integration. Our method enhances the capability of capturing detailed features across branches of various resolutions, especially in low-resolution branches, thereby improving the model’s sensitivity to certain keypoints (like ankles).

Difference with SENet [21]. Both model use SE block as component to adaptively recalibrates channel-wise feature responses. But they differ in the following aspects: SENet focuses on learning inter-channel dependencies, where the input at each stage is derived from the resolution-specific streams of the previous stage. However, our DEM employs downsampled higher-resolution features from preceding stages combined with aggregated branches at the current resolution. We posit that integrating higher-resolution features from upper stages facilitates the aggregation of feature hierarchies absent in the current resolution, thereby enhancing spatial encoding quality and strengthening the representational capacity of CNNs. Additionally, the aggregated feature information aids in recalibrating overlooked or missing detail information through feature reweighting.

## 4 Experiments

To verify the impact of our method on the accuracy of keypoint localization in scale variations, we compared with other related methods on two datasets, MS COCO dataset [26] and MPII Human Pose dataset [27].

### 4.1 COCO keypoint detection

#### 4.1.1 Dataset and evaluation metric.

The COCO dataset [26] comprises over 200,000 images and 250,000 annotated instances of humans with 17 keypoints, which is useful for learning complex models capable of precise localization. We utilize the COCO 2017 dataset, which is split into training, validation, and test sets at an approximate 11:1:4 ratio. The evaluation metrics adopted are the average precision (AP) and average recall (AR), both based on object keypoint similarity (OKS): $\begin{array}{c} OKS\;=\frac{\sum_{i}\text{exp}\left(\frac{d_{i}^{2}}{{2Sk}_{i}^{2}}\right)\delta \left(v_{i}>0\right)}{\sum_{i}\delta \left(v_{i}>0\right)} \end{array}$ [26].

#### 4.1.2 Training.

The network is trained on 4 NVIDIA GeForce RTX 2080 Ti GPUs with batch size 32 per GPU. We use the Adam optimizer [28] and the base learning rate is 1e-3, it drops to 1e-4 at the 170th and 1e-5 at the 200th epochs. There are 210 epochs in total.

Following [9], we use the same human detection box cropping method, each image is resized to 384×288. Data augmentation includes scale(±35%), rotation(±45degrees), flipping and half body data augmentation.

#### 4.1.3 Testing.

Consistent with the backbone network, we used faster-RCNN [29] as the human detector. In line with hourglass [22], we averaged the predicted heatmaps from the flipped images with those from the original images to estimate the joint positions, then taking the location offset by one quarter of the distance from the highest to the second-highest response to determine the final predicted position.

#### 4.1.4 Results on the validation set.

Table 1. reported the results of our and other related methods. Our DE-HRNet-32 trained from the model pretrained on the ImageNet with the input size 384×288, achieves a 76.4 AP score, outperforming other methods with the same input size. Compared to SimpleBaseline [31], our network improves AP by 1.9 points, and the GFLOPs of our network and the number of parameters is much lower and less than half. Compared to HRNet-32 and HRNet-48, our network DE-HRNet-32 and DE-HRNet-48, with slightly larger model size and slightly higher complexity, achieves 0.6 and 0.3 gain, respectively.

**Table 1:** Comparisons on the COCO validation set. #Params and FLOPS are calculated only for the method.

Method	Backbone	Input size	#Param	GFLOPs	AP	${AP}^{50}$	${AP}^{75}$	${AP}^{M}$	${AP}^{L}$	AR
HRFormer [6]	HRFormer-S	384×288	7.8M	6.2G	75.6	90.3	82.2	71.6	82.5	80.7
HRFormer [6]	HRFormer-B	384×288	43.2M	26.8G	**77.2**	**91.0**	**83.6**	**73.2**	**84.2**	**82.0**
HRNet+dark [30]	HRNet-W48	384×288	63.6M	32.9G	76.6	90.7	82.8	72.7	83.9	81.5
SimpleBaseline [31]	ResNet-152	384×288	68.6M	35.6G	74.3	89.6	81.1	70.5	79.7	79.7
HRNet [5]	HRNet-W32	384×288	28.5M	16.0G	75.8	90.6	82.7	71.9	82.8	81.0
HRNet [5]	HRNet-W48	384×288	63.6M	32.9G	**76.3**	**90.8**	**82.9**	**72.3**	**83.4**	**81.2**
Ours	HRNet-W32	384×288	33.6M	17.7G	76.4 (+0.6)	**90.6**	83.2	72.6	83.5	81.5
Ours	HRNet-W48	384×288	74.8M	36.6G	**76.6** (+0.3)	90.4	**83.3**	**73.0**	**83.6**	**81.6**

#### 4.1.5 Results on the test-dev set.

Table 2. reported the results of our and other state-of–the-art methods. First, our method is better compared to bottom-up approaches designed to address scale variation challenges. Second, our DE-HRNet achieves 75.6 AP on the test set, showing comparable gains (+0.7) to the validation set improvements (+0.6), which substantiates its generalization capability across different data distributions. Notably, under the 384×288 input setting, the performance gap between our lightweight variant (33.6M parameters) and the full model (74.8M) becomes statistically insignificant, suggesting that model compression can be strategically applied in resolution-constrained scenarios. The sole limitation of our method compared to the original HRNet lies in its increased model size (by nearly one-fifth in parameters) and elevated computational complexity (by approximately one-tenth in FLOPs).

**Table 2:** Comparisons on the COCO test-dev set. The bottom-up methods use multi-scale testing.

Method	Backbone	Input size	#Params	GFLOPs	AP	${AP}^{50}$	${AP}^{75}$	${AP}^{M}$	${AP}^{L}$	AR
	Bottom-up methods									
Hourglass [15]	—	512 × 512	277.8M	206.9G	63.0	85.7	68.9	58	70.4	—
HrHRNet [4]	HRNet-W48	640 × 640	63.8M	154.3G	70.5	89.3	77.2	66.6	75.8	—
HrHRNet+SWAHR [7]	HRNet-W48	640 × 640	63.8M	154.6G	**72.0**	**90.7**	**78.8**	**67.8**	**77.7**	—
	Top-down methods									
CPN (ensemble) [12]	ResNet-Inception	384 × 288	—	—	73.0	91.7	80.9	69.5	78.1	79.0
SimpleBaseline [31]	ResNet-152	384 × 288	68.6M	35.6 G	73.7	91.9	81.1	70.3	80.0	79.0
CFN [32]	CFN	448 × 448	—	—	72.6	86.1	69.7	**78.3**	64.1	—
MSPN [33]	4×Res-50	384 × 288	71.9M	58.7G	76.1	93.4	83.8	72.3	81.5	81.6
RSN [8]	4×RSN-50	384 × 288	111.8M	65.9 G	**78.6**	**94.3**	**86.6**	75.5	**83.3**	**83.8**
HRFormer [6]	HRFormer-B	384 × 288	43.2M	26.8 G	**76.2**	92.7	83.8	72.5	82.3	81.2
TokenPose [34]	TokenPose-L	384 × 288	29.8M	22.1 G	75.9	92.3	83.4	72.2	82.1	80.8
HRNet+dark [30]	HRNet-W48	384 × 288	63.6M	32.9 G	76.2	92.5	83.6	72.5	82.4	81.1
HRNet + UDP [35]	HRNet-W48	384 × 288	63.8M	33.0 G	**76.5**	92.7	84.0	73.0	82.4	81.6
HRNet [5]	HRNet-W32	384 × 288	28.5M	16.0 G	74.9	92.5	82.8	71.3	80.9	80.1
HRNet [5]	HRNet-W48	384 × 288	63.6M	32.9 G	**75.5**	92.5	83.3	71.9	81.5	80.5
Ours	HRNet-W32	384 × 288	33.6M	17.7 G	75.6 (+0.7)	92.4	83.3	72.1	81.6	80.7
Ours	HRNet-W48	384 × 288	74.8M	36.6 G	**75.7** (+0.2)	92.3	83.2	72.2	81.7	80.9

### 4.2 MPII human pose estimation

#### 4.2.1 Dataset, training and evaluation metric.

The MPII Human Pose dataset [27] contains 40k person samples, each image labelled with 16 joints. The dataset is split into training and validation sets in a ratio of approximately 7:6. Following [5], we adopt the same training, testing and data augmentation strategies (such as the input size is cropped to 256×256).We employ the standard Percentage of Correct Keypoints (PCK) [27] metric. PCK reports measures the proportion of detected keypoints lying within a normalized distance threshold from their ground-truth locations. To ensure evaluative fairness, all performance metrics are reported using PCKh@0.5, a standardized evaluation protocol where the matching threshold corresponds to 50% of the head segment length.

#### 4.2.2 Results on the validation set.

Table 3 reports the results of our method and other methods on the MPII valid set. To compare with other method, our results are rounded upwards to preserve one decimal place. Our method achieves 90.7 PCKh@0.5 score, Compared to HRNet and SimpleBaseline, achieves 0.4 and 0.5 gain, respectively. In line with HRNet, we also training our method (DE-HRNet-w48), its accuracy not significantly different.

**Table 3:** Comparisons of PCK@0.5 score on the MPII valid set.

Arch	Head	Shoulder	Elbow	Wrist	Hip	Knee	Ankle	Mean
SimpleBase-line [31]	96.8	95.6	90.1	86.2	89.7	86.9	82.9	90.2
HRNet [5]	97.1	95.9	90.3	86.4	89.1	**87.1**	83.3	90.3
HRNet+dark [30]	97.2	95.9	**91.2**	86.7	89.7	86.7	**84.0**	90.6
Ours	**97.6**	**96.3**	90.9	**87.3**	**90.1**	86.7	82.9	**90.7**

### 4.3 Ablation study

In this subsection, through systematic ablation studies on the MPII human pose benchmark, we quantify the contribution of individual architectural components to keypoint prediction consistency, with all evaluations strictly adhering to the 256×256 input protocol.

Table 4 illustrates the effects of two critical components in our method, dySample and the Detail Enhancement Module (DEM), on multi-joint prediction. We progressively integrated the DEM and dynamic sampler (dySample) into the HRNet-W32 baseline through stepwise ablation studies. As shown in Table 4 (b), replacing only the dynamic sampler (dySample) yields 90.5 PCKh@0.5, achieving a 0.2 point improvement over the baseline HRNet-W32. Table 4 (c) demonstrates that integrating the Detail Enhancement Module (DEM) exclusively into parallel multi-resolution branches attains 90.4 PCKh@0.5, corresponding to a 0.1 point gain compared to the original HRNet-W32. Table 4 reveals two critical observations: First, the substitution with dySample effectively reduces detail loss through adaptive feature resampling, which significantly improves the model’s capability in multi-scale joint prediction. Second, the experimental results of DEM validate that our designed Detail Enhancement Module effectively processes multi-scale features while augmenting reconstructed local details, thereby enhancing the overall performance of human pose estimation.

**Table 4:** Ablation study of DE-HRNet’s components on the MPII valid set.

Methods	HRNet	dySample	DEM (SE block)	Head	Shoulder	Elbow	Wrist	Hip	Knee	Ankle	Mean
(a)	√			97.1	95.9	90.3	86.4	89.1	**87.1**	83.3	90.3
(b)	√	√		96.9	96.0	90.9	86.6	89.5	86.6	**83.6**	90.5
(c)	√		√	97.4	96.2	90.7	86.9	89.8	86.9	82.8	90.4
(d)	√	√	√	**97.6**	**96.3**	**90.9**	**87.3**	**90.1**	86.7	82.9	**90.7**

Additionally, we validate the impact of SENet and ECANet by replacing DEM. The results are shown in Table 5. The experimental results demonstrate that incorporating either SE blocks or ECA blocks leads to degraded prediction accuracy across all body joints. We therefore propose the Detail Enhancement Module (DEM) by reconstructing these baseline attention components, which improves multi-scale keypoint detection accuracy.

**Table 5:** The ECA block and SE block components on the MPII valid set.

Methods		Head	Shoulder	Elbow	Wrist	Hip	Knee	Ankle	Mean
(a)	HRNet [9]	**97.1**	95.9	90.3	**86.4**	89.1	**87.1**	83.3	90.3
(b)	HRNet + dySample	96.9	96.0	90.9	86.6	**89.5**	86.6	**83.6**	**90.5**
(c)	HRNet + dySample + SE block	96.6	**96.0**	**90.6**	86.3	89.3	86.1	82.9	90.2
(d)	HRNet + dySample + ECA block	97.0	95.7	90.4	85.9	88.9	86.3	82.7	90.0
(e)	HRNet + DEM (ECA block)	97.0	96.0	**90.8**	86.3	89.6	**87.2**	**83.5**	**90.5**
(f)	HRNet + DEM (SE block)	**97.4**	**96.2**	90.7	**86.9**	**89.8**	86.9	82.8	90.4
(g)	HRNet + dySample + DEM (ECA block)	97.1	96.0	**91.2**	86.6	90.0	**86.9**	82.8	90.5
(h)	HRNet + dySample + DEM (SE block)	**97.6**	**96.3**	90.9	**87.3**	**90.1**	86.7	**82.9**	**90.7**

### 4.4 Discussions

Table 2 compares DE-HRNet with recent HRNet variants across two technical directions: Transformer-based adaptations (HRFormer [6] and TokenPose [34]), Quantization-error-aware frameworks (DARK [30] and UDP [35]). In addition, there are also some advanced CNN architectures (RSN [8] and MSPN [33]). While DE-HRNet shows modest accuracy gaps compared to these specialized approaches (e.g., −0.6% AP against HRFormer on COCO test-dev). Unlike transformer-based methods [6,34] that capture global dependencies at higher computational cost, our convolutional enhancement module prioritizes local feature refinement for speed (Ours 17.7G vs HRFormer 26.8G GFLOPs). Unlike quantization error correction methods [30,35] that primarily operate through pre-processing or post-processing stages, our approach enhances the network’s capability of capturing multi-scale joint features through embedded convolutional modules. While this design choice inevitably increases parameter count and model complexity, it fundamentally addresses scale variation challenges from the feature learning perspective. Meanwhile, compared to large convolutional architectures [8,33], our HRNet-based optimized method demonstrates distinct efficiency advantages: While our approach currently exhibits modest performance gaps (approximately 0.5% AP difference on COCO test-dev), it requires only 47% of the computational resources and 30% of the parameters compared to MSPN [33], making it more suitable for deployment-constrained scenarios. Therefore, our method DE-HRNet improves efficiency in addressing the challenge of scale variations.

In the ablation experiments of Table 4 regarding the decreased prediction accuracy for knee or ankle joints, we conducted further experimental analysis and obtained Table 5. We analyzed the causes of accuracy degradation through the changes in joint accuracy in configurations (a), (b), (e), and (g). Among these: (a) is the HRNet baseline, (b) is the HRNet baseline integrated with dySample, (e) is the HRNet baseline integrated with DEM and SE block, (g) is the HRNet baseline integrated with dySample, DEM, and SE block. Taking the knee joint accuracy scores as an example: (a) achieves 87.1, (b) 86.6, (e) 86.9, and (g) 86.7. The data suggest that the introduced contextual information from DEM may interfere with the detail enhancement for knee joints, leading to decreased prediction scores. Additionally, dySample [17], derived from point sampling, maintains depth values in flat regions while processing gradually changing depth values. We infer that dySample [17] is less effective than adjacent upsampling when handling features with complex local depth variations.

Compared to the above methods, our current analysis does not specifically address the robustness of our method under special scenarios such as occlusion or extreme pose variations. But the COCO keypoint dataset [26] provides naturally challenging images with diverse human poses, varying body scales, and complex occlusion patterns.

## 5 Conclusion

In this paper, we aim to enhance the precision of anatomical keypoint localization under scale variations. we propose a novel Detail-Enhanced High-Resolution Network (DE-HRNet), an architectural extension of HRNet tailored for human pose estimation. Our innovation stems from two advancements: First, we Detail Enhancement Module (DEM) that facilitates multi-level feature fusion for preserving localized texture patterns in high-resolution streams. Second, we introduce dynamic sampler – dySample which is replacing neighbor interpolation upsampling, which mitigates representational degradation during resolution recovery.

Our method are suitable to same position sensitive vision applications, such as sports analytics or autonomous driving. In addition, our method still has limitations: the analysis under extreme poses or heavy occlusion is insufficient, and the model contains a large number of parameters. In future work, we will explore Transformer-based approaches to address extreme poses and occlusions, apply pruning and other compression techniques for model lightweighting. Additionally, we plan to explore 3D pose estimation, which could resolve many challenges (scale variations, illumination changes, occlusions, and complex backgrounds) in human pose estimation.
